# Mediterranean diet adherence is associated with lower dementia risk, independent of genetic predisposition: findings from the UK Biobank prospective cohort study

**DOI:** 10.1186/s12916-023-02772-3

**Published:** 2023-03-14

**Authors:** Oliver M. Shannon, Janice M. Ranson, Sarah Gregory, Helen Macpherson, Catherine Milte, Marleen Lentjes, Angela Mulligan, Claire McEvoy, Alex Griffiths, Jamie Matu, Tom R. Hill, Ashley Adamson, Mario Siervo, Anne Marie Minihane, Graciela Muniz-Tererra, Craig Ritchie, John C. Mathers, David J. Llewellyn, Emma Stevenson

**Affiliations:** 1 Human Nutrition & Exercise Research Centre, Centre for Healthier Lives, Population Health Sciences Institute, Newcastle University, Newcastle Upon Tyne, UK; 2 College of Medicine and Health, University of Exeter, Exeter, UK; 3 Edinburgh Dementia Prevention, Centre for Clinical Brain Sciences, University of Edinburgh, Edinburgh, UK; 4 Institute for Physical Activity and Nutrition, School of Exercise and Nutrition Sciences, Deakin University, Geelong, VIC Australia; 5 School of Medical Sciences, Örebro University, Örebro, Sweden; 6 Nutrition Measurement Platform, MRC Epidemiology Unit, University of Cambridge, Cambridge, UK; 7 Centre for Public Health, The Institute for Global Food Security, Queen’s University Belfast, Belfast, UK; 8 School of Health, Leeds Beckett University, Leeds, UK; 9 School of Life Sciences, Queen’s Medical Centre, The University of Nottingham Medical School, Nottingham, UK; 10 Nutrition and Preventive Medicine, Norwich Medical School, University of East Anglia, Norwich, UK; 11 Norwich Institute of Health Ageing (NIHA), Norwich, UK; 12 Heritage College of Osteopathic Medicine, Ohio University, Athens, OH USA; 13 Alan Turing Institute, London, UK

**Keywords:** Dementia, Alzheimer’s, Mediterranean diet, Genetics, Polygenic risk, Risk factors, UK Biobank

## Abstract

**Background:**

The identification of effective dementia prevention strategies is a major public health priority, due to the enormous and growing societal cost of this condition. Consumption of a Mediterranean diet (MedDiet) has been proposed to reduce dementia risk. However, current evidence is inconclusive and is typically derived from small cohorts with limited dementia cases. Additionally, few studies have explored the interaction between diet and genetic risk of dementia.

**Methods:**

We used Cox proportional hazard regression models to explore the associations between MedDiet adherence, defined using two different scores (Mediterranean Diet Adherence Screener [MEDAS] continuous and Mediterranean diet Pyramid [PYRAMID] scores), and incident all-cause dementia risk in 60,298 participants from UK Biobank, followed for an average 9.1 years. The interaction between diet and polygenic risk for dementia was also tested.

**Results:**

Higher MedDiet adherence was associated with lower dementia risk (MEDAS continuous: HR = 0.77, 95% CI = 0.65–0.91; PYRAMID: HR = 0.86, 95% CI = 0.73–1.02 for highest versus lowest tertiles). There was no significant interaction between MedDiet adherence defined by the MEDAS continuous and PYRAMID scores and polygenic risk for dementia.

**Conclusions:**

Higher adherence to a MedDiet was associated with lower dementia risk, independent of genetic risk, underlining the importance of diet in dementia prevention interventions.

**Supplementary Information:**

The online version contains supplementary material available at 10.1186/s12916-023-02772-3.

## Background

Preventing dementia is a global public health priority due to the enormous and growing societal cost of this condition [1]. A key strategy to reduce dementia incidence is the identification of modifiable risk factors that can be targeted by personalized or public health interventions. These modifiable risk factors, in combination with genetic risk, play a key role in determining individual risk of Alzheimer’s disease and other forms of dementia [2–4]. Diet is an important modifiable risk factor for dementia that could be targeted for disease prevention and risk reduction [5, 6]. Healthier dietary patterns, especially the Mediterranean diet (MedDiet), have been proposed as a strategy to reduce dementia risk [7, 8]. Recent systematic [9] and umbrella [10] reviews have suggested higher adherence to the MedDiet may reduce cognitive decline, although evidence for a protective role of the MedDiet against dementia risk is inconsistent [11–16]. As most prior studies have been conducted in relatively small cohorts (*n* = 1000–6000) with limited numbers of dementia cases (*n* = 20–400), additional investigations which leverage large population-based cohorts are warranted. There is also currently no gold standard assessment of MedDiet adherence, and some variability in study findings may therefore be due to different dietary assessment methods and scoring systems [17]. Therefore, studies comparing different MedDiet scores directly and their associations with dementia risk are needed.

A healthy diet might also mitigate individual genetic risk for dementia. Previous studies exploring gene-diet interactions are limited, have reported inconsistent results, and, typically, focus on *APOE* genotype as the sole measure of genetic risk [13, 18–20]. Polygenic risk scores combining information from multiple weighted (i.e., according to the strength of their association with dementia) risk alleles predict incident all-cause dementia [21, 22] and are an important advance in facilitating in-depth exploration of potential gene-diet interactions.

The purpose of this study was to investigate associations between MedDiet adherence and dementia incidence in a large prospective cohort study, and to explore the interaction between diet and genetic risk for dementia.

## Methods

### Study population and design

The UK Biobank is an ongoing, multi-centre prospective cohort study of over half a million participants, that provides a resource for investigating the determinants of disease in middle and older age [23]. The design and methods of this study have been described elsewhere [24]. Briefly, between 2006 and 2010, men and women aged 40–69 years were recruited from across England, Scotland and Wales using National Health Service (NHS) patient registers. Participants attended one of 22 assessment centres where they completed a touchscreen questionnaire, verbal interview, and provided measures of physical function alongside biological samples. Subsequently, participants were invited to complete additional measures, including enhanced dietary assessments, imaging, and assessment of multiple health-related outcomes. UK Biobank also includes linkage to electronic healthcare records (death, cancer, inpatient and primary care records) for disease ascertainment. Ethical approval for the UK Biobank study was provided by the North West–Haydock Research Ethics Committee (REC reference: 16/NW/0274), and all participants provided electronic signed consent. The current study included participants who self-reported a racial/ ethnic background of white British, Irish or other white, were aged ≥ 60 years at recruitment with genetic data, appropriate dietary data (self-reported atypical dietary reports were excluded) and were not missing data for any of the included covariates (Additional file 1, Fig. S1).

### Dietary assessment and calculation of Mediterranean diet scores

The Oxford WebQ is a web-based, self-administered 24-h dietary assessment tool, validated for use in large-scale observational studies [25, 26]. This tool collects information about the consumption of 206 types of foods and 32 types of drinks during the previous 24-h period, with participants selecting the number of standard portions for each item that they consumed. Participants recruited between April 2009 and September 2010 completed the Oxford WebQ as part of their baseline assessment centre visits. In addition, between February 2011 and June 2012, participants were invited to complete the Oxford WebQ assessment via their home computer every three to four months, up to a total of five assessments (including the baseline assessment). Consistent with previous investigations [17, 27], we energy-adjusted the dietary data (2000 kcal/d) for each time point via the residuals method to allow evaluation of diet quality independent of diet quantity [28]. Data were then averaged across all available time points (minimum 1, maximum 5) for each participant prior to calculation of MedDiet scores.

We quantified MedDiet adherence using two separate scores: the MedDiet Adherence Screener (MEDAS) score, and the MedDiet PYRAMID score. These scores define MedDiet adherence in different ways (e.g., using different dietary targets and food components) and therefore may differ in terms of their association with dementia.

### MEDAS score

The MEDAS is a 14-point score developed as part of the Prevención con Dieta Mediterránea (PREDIMED) trial [29] that has been used widely in trials and observational studies [8, 30]. The MEDAS has been validated for use in the UK (the UK-validated version of the MEDAS was used to develop our MEDAS scores in this study) [31] and endorsed for use as a rapid diet assessment screening tool in clinical practice by the American Heart Association [32]. The MEDAS is conventionally calculated with a binary evaluation for each of the 14 food components, with one point awarded if the participant’s consumption meets a pre-defined cut-off (e.g., intake of a specific amount of vegetables), and zero points if they do not. The total possible score ranges from 0–14 points. We have shown previously that using the same dietary targets but implementing an alternative continuous scoring system using linear equation principles (y = ax + b, in which y is the number of points scored between 0 and 1, a is the slope and b is the intercept), in which points are awarded between zero and one depending upon proximity to the dietary targets, increases the sensitivity of this score in detecting differences in diet quality [17]. Therefore, this score, referred to here as the MEDAS continuous score, was used for the primary analyses in the present study. As a hypothetical example to illustrate the difference between the MEDAS and MEDAS continuous scores, an individual with a daily vegetable intake of 295 g or ~ 1.5 * 200 g servings of vegetables would be awarded 0 points for this specific MedDiet component for the MEDAS score, as they have not achieved the dietary target of 2 servings (i.e., 400 g) vegetable intake per day. By contrast, according to the MEDAS continuous score, this individual would be awarded ~ 0.74 points (y = 0.5 * 1.475 + 0 = 0.7375 points), based around how close they are to the specific dietary target (i.e., ~ 3/4 of the way towards achieving the dietary target). We repeated the analysis using the conventionally-scored MEDAS as a sensitivity analysis.

Both the MEDAS and MEDAS continuous scores award points for use of olive oil as the main culinary fat and, separately, for consumption of a target amount (4 or more tablespoons per day) of olive oil. Although we were able to determine use of olive oil as a culinary fat and to award points for consumption (1 point) or non-consumption (0 points) accordingly, it was not possible to determine the amount of olive oil consumed from the available dietary data, limiting the maximum possible scores for the MEDAS and MEDAS continuous to 13 points in this study.

### PYRAMID score

The PYRAMID score is a 15-point MedDiet score used widely in epidemiological studies [9, 17, 27]. Each of the 15 individual components are coded on a continuous basis with scores ranging from zero to one (26). Further details of the calculation of each MedDiet score is provided in Additional file 1, Tables S1 and S2. For both MedDiet scores, higher values reflect greater adherence to the MedDiet.

### Polygenic risk score

To estimate genetic risk of dementia, we used the polygenic risk score developed by Lourida and colleagues, who demonstrated that higher values of this score are associated with higher all-cause dementia risk in the UK Biobank cohort [22]. The score was based on a genome-wide association study of individuals of European ancestry [33]. Therefore, the current analysis was restricted to individuals who self-reported a racial/ ethnic background of white British, Irish or other white (who constitute > 90% of the UK Biobank cohort). For the primary analyses, the polygenic risk score was divided into quintiles, and participants were categorised into low (quintile 1), medium (quintiles 2–4) and high (quintile 5) risk groups. A total of 249,273 independent genetic variants were used to create the polygenic risk score. Further details of the polygenic risk score creation and this approach can be found elsewhere [22].

### Dementia outcome ascertainment

All-cause incident dementia cases were ascertained using data linkage to hospital inpatient records and death registries. Diagnoses were recorded using the International Classification of Diseases (ICD) coding system [34]. Participants with a primary or secondary diagnosis of dementia were identified from hospital records or underlying/contributory cause of death from death registries using relevant ICD-9 and ICD-10 codes (Additional file 1, Table S3.). We used the censoring dates recommended by UK Biobank for death data and hospital inpatient data. These are the dates up to which the data is estimated to be over 90% complete in England, Scotland and Wales separately. At the time of analysis, the recommended censoring dates were 31^st^ March, 2021 for England and Scotland, and 28^th^ February, 2018 for Wales. The mean (SD) and median (interquartile range) follow up was 9.1 (1.7) and 9.3 (8.8–9.7) years, respectively. Follow up time was calculated from the most recent eligible dietary report used for MedDiet score creation and either the date of first dementia diagnosis, death, loss to follow-up, or censoring date, whichever was the earliest.

### Statistical analysis

All analyses were conducted in SPSS version 27. Baseline characteristics of the analytic sample, stratified by dementia status, were summarised as mean ± SD for continuous variables and as percentages for categorical variables. Cox proportional hazard regression models were used to examine the association between MedDiet adherence and time to incident all-cause dementia, with the duration of follow-up in years used as the timescale. We also explored the association between the polygenic risk score and dementia incidence, to confirm the previously reported associations between these variables in this cohort [22]. The possible interaction between MedDiet adherence and polygenic risk for dementia was investigated by including an interaction term, with both variables expressed continuously.

Analyses were adjusted simultaneously for: age, sex, socioeconomic status (Townsend Index categorised as low [quintile 1], moderate [quintiles 2–4], high [quintile 5] deprivation), education (higher [college/university/other professional qualification], vocational [NVQ/HND/HNC], upper secondary [A-levels], lower secondary [O-levels/GCSEs /CSEs] or none), smoking status (never, past, current), typical sleep duration (< 7, 7–8, > 8 h), physical activity (international physical activity questionnaire [IPAQ] group, categorised as low, medium, high), energy intake (kcal/d), third-degree relatedness of individuals in the sample, and the first 20 principal components of ancestry. Models which included the polygenic risk score were additionally adjusted for the number of alleles included in the score, to account for SNP-level variation [22]

### Sensitivity analyses

Sensitivity analyses were performed to test the robustness of associations between MedDiet adherence and dementia incidence. First, we used the conventional binary MEDAS score. Secondly, we included participants with a minimum of two, 24-h diet recalls to provide a more stringent measure of habitual dietary intake [26]. Thirdly, we excluded participants with 24-h recalls with extreme energy intakes (defined as < 800 or > 4200 kcal/d for males and < 600 or > 3500 kcal/d for females) [35]. Fourth, to assess whether any individual components of the MedDiet drove the observed associations, we repeated the analyses after sequentially removing each MedDiet component from the total score. Fifth, in consideration of the potential for reverse causality, we repeated the primary analyses after excluding participants with less than 2- and less than 5-years of follow-up, respectively. Sixth, we repeated the analyses including potential mediators individually; stroke history (yes/no for any type of stroke diagnosed prior to dementia diagnosis or end of follow-up for those who remained dementia-free), self-reported depressive symptoms (yes/no for reporting feeling down/depressed/hopeless on ‘several days’, ‘more than half the days’ or ‘nearly every day’), and body mass index (BMI) category (< 25, 25–29.9, > 30 kg/m^2^). Seventh, as an alternative method of exploring whether associations between MedDiet adherence and dementia risk were influenced by polygenic risk score, we conducted stratified analyses exploring associations between MedDiet adherence and dementia risk in low, medium and high genetic risk categories. Eighth, we investigated the interaction between MedDiet adherence and genetic risk, with genetic risk defined by Apolipoprotein E (*APOE*) genotype only (a more common but less comprehensive measure of genetic risk, which may be easier to apply in clinical practice). *APOE* ε4 carriers were defined as higher risk, whilst non-carriers were defined as lower risk. Nineth, to evaluate the influence of missing data, we repeated analyses following imputation of missing dietary and covariate data using multiple imputations by chained Eqs. (70 imputations, 20 iterations) [36]. We included all analytic variables (covariates and outcome data) as predictors in the model. In addition, we created abbreviated MedDiet scores using dietary data from the UK Biobank touchscreen questionnaire (data available for all participants) which were used as auxiliary variables in the imputation model. Tenth, we carried out separate analyses for fatal and non-fatal cases of dementia. Eleventh, we conducted stratified analyses in individuals with higher (college/university/other professional qualification) and lower (vocational, upper secondary, lower secondary, and none) education levels.

## Results

### Cohort characteristics

A total of 502,536 participants underwent baseline assessment as part of the UK Biobank study, of whom 60,298 participants were included in this analysis (See Additional file 1, Fig. S1 for the study inclusion flow diagram). Baseline characteristics of the participants, stratified by level of MedDiet adherence (low, medium, and high MEDAS continuous scores), are provided in Table 1. During a mean (SD) follow up of 9.1 (1.7) years and a total of 549,999 person years, there were 882 cases of incident all-cause dementia. Participants with a higher MedDiet adherence according to the MEDAS continuous score were more likely to be female, have a BMI within the healthy range (< 25 kg/m^2^), have a higher educational level, and be more physically active than those with lower MedDiet adherence. The mean MEDAS continuous and PYRAMID scores in this cohort were 6.1 ± 1.7 and 7.5 ± 1.8, respectively.

**Table 1:** Participant characteristics of the analytic sample of UK Biobank, stratified by level of MedDiet adherence according to the MEDAS continuous score

	Total (*n* = 60,298)	MEDAS continuous low (0–5.3) (*n* = 19,393, 32.2%)	MEDAS continuous medium (> 5.3–6.8) (*n* = 20,120, 33.4%)	MEDAS continuous High (> 6.8) (*n* = 20,785, 34.5%)
Age (mean ± SD), years	63.8 ± 2.7	63.9 ± 2.8	63.9 ± 2.8	63.8 ± 2.7
Sex				
Male	31,066 (51.5%)	11,252 (58%)	10,252 (51.0%)	9562 (46.0%)
Female	29,232 (48.5%)	8141 (42.0%)	9868 (49.0%)	11,223 (54.0%)
BMI^a^ (kg/m^2^)				
< 25	20,780 (34.5%)	5526 (28.5%)	6826 (33.9%)	8428 (40.5%)
25–29.9	27,154 (45.1%)	8835 (45.6%)	9259 (46.0%)	9060 (43.6%)
> 30	12,229 (20.3%)	4984 (25.7%)	3994 (19.9%)	3251 (15.6%)
Education				
Higher	33,291 (55.2%)	8799 (45.4%)	11,162 (55.5%)	13,330 (64.1%)
Vocational	6143 (10.2%)	2391 (12.3%)	2036 (10.1%)	1716 (8.3%)
Upper secondary	3377 (5.6%)	1015 (5.2%)	1132 (5.6%)	1230 (5.9%)
Lower secondary	9270 (15.4%)	3387 (17.5%)	3161 (15.7%)	2722 (13.1%)
Other	8217 (13.6%)	3801 (19.6%)	2629 (13.1%)	1787 (8.6%)
Socioeconomic status^b^				
1 (least deprived)	14,375 (23.8%)	4279 (22.6%)	4987 (24.8%)	5009 (24.1%)
2–4	38,142 (63.3%)	12,370 (63.8%)	12,774 (63.5%)	12,998 (62.5%)
5 (most deprived)	7781 (12.9%)	2644 (13.6%)	2359 (11.7%)	2778 (13.4%)
Smoking status				
Never	30,686 (50.9%)	9645 (49.7%)	10,400 (51.7%)	10,641 (51.2%)
Previous	26,157 (43.4%)	8197 (42.3%)	8725 (43.4%)	9235 (44.4%)
Current	3455 (5.7%)	1551 (8.0%)	995 (4.9%)	909 (4.4%)
Typical sleep duration				
< 7/hours	12,402 (20.6%)	4252 (21.9%)	4088 (20.3%)	4062 (19.5%)
7–8 h	42,813 (71%)	13,287 (68.5%)	14,346 (71.3%)	15,180 (72.0%)
> 8 h	5083 (8.4%)	1854 (9.6%)	1686 (8.4%)	1543 (7.4%)
Physical activity levels^c^				
Low (least active)	9921 (16.5%)	3786 (19.5%)	3372 (16.8%)	2763 (13.3%)
Moderate	26,021 (43.2%)	8339 (43.0%)	8727 (43.4%)	8955 (43.1%)
High (most active)	24,356 (40.4%)	7268 (37.5%)	8021 (39.9%)	9067 (43.6%)
Genetic risk category^d^				
Low	12,703 (21.1%)	4044 (20.9%)	4104 (20.4%)	4555 (21.9%)
Medium	36,085 (59.8%)	11,594 (59.8%)	12,051 (59.9%)	12,440 (59.9%)
High	11,510 (19.1%)	3755 (19.4%)	3965 (19.7%)	3790 (18.2%)
Dementia incidence				
Incident dementia	882 (1.5%)	336 (1.7%)	301 (1.5%)	245 (1.2%)
No incident dementia	59,416 (98.5%)	19,057 (98.3%)	19,819 (98.5%)	20,540 (98.8%)

^a^BMI data available in *n* = 60,163 participants (incident dementia *n* = 876, no incidence dementia *n* = 59,287)

^b^Socioeconomic status includes categories derived from Townsend deprivation index, with quintiles 1 = low (least deprived), 2–4 = medium, 5 = high (most deprived)

^c^Self-reported physical activity levels according to the International Physical Activity Questionnaire (IPAQ)

^d^Genetic risk category, with quintiles 1 = low, 2–4 = medium, 5 = high

### Mediterranean diet adherence and risk of incident dementia

**Fig. 1:**
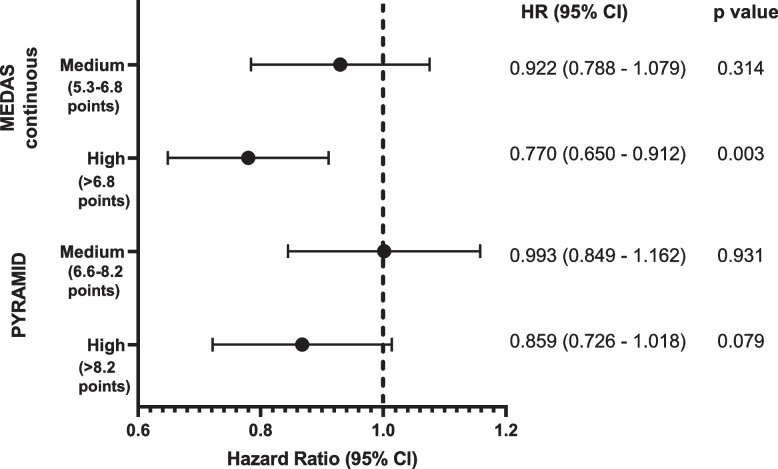
Association between MedDiet adherence and risk of dementia (*n* = 60,298, including 882 dementia cases). MedDiet adherence level was split into tertiles, with the dashed line reflecting the low MedDiet adherence reference group for each MedDiet score

Higher adherence to the MedDiet was associated with 4.2–6.9% lower risk for dementia for the MEDAS continuous (HR per one point increase in MedDiet score: 0.931; 95% CI: 0.895–0.969; *p* < 0.001) and PYRAMID (HR per one point increase in MedDiet score: 0.958; 95% CI: 0.922–0.996; *p* = 0.031) scores. When divided into tertiles, relative to low MedDiet, high but not moderate adherence was associated with lower dementia risk (Fig. 1**)**.

The absolute risk of dementia in the low, medium and high MedDiet adherence groups defined by the MEDAS continuous score was 1.73%, 1.50% and 1.18%, respectively. Moderate and high MedDiet adherence groups had 0.23% (95% CI: -0.01–0.49%) and 0.55% (95% CI: 0.32–0.79%) lower absolute risk of dementia than those in the low MedDiet adherence group. Meanwhile, the absolute risk of dementia in the low, medium and high MedDiet adherence groups defined by the PYRAMID score was 1.67%, 1.53%, and 1.21%, respectively. Moderate and high MedDiet adherence groups had 0.14% (95% CI: -0.1–0.39%) and 0.46% (95% CI: 0.23–0.69%) lower absolute risk of dementia than those in the low MedDiet adherence group.

### Mediterranean diet adherence, genetic risk and dementia incidence

A higher polygenic risk score was associated with greater risk for dementia (HR: 1.224, 95% CI: 1.102–1.360; *p* < 0.001). There was no significant interaction between polygenic risk for dementia and MedDiet adherence defined by the MEDAS continuous (HR: 1.036, 95% CI: 0.977–1.076; *p* = 0.070) or PYRAMID (HR: 1.011; 95% CI: 0.974–1.049; *p* = 0.572) scores.

### Sensitivity analyses

**Fig. 2:**
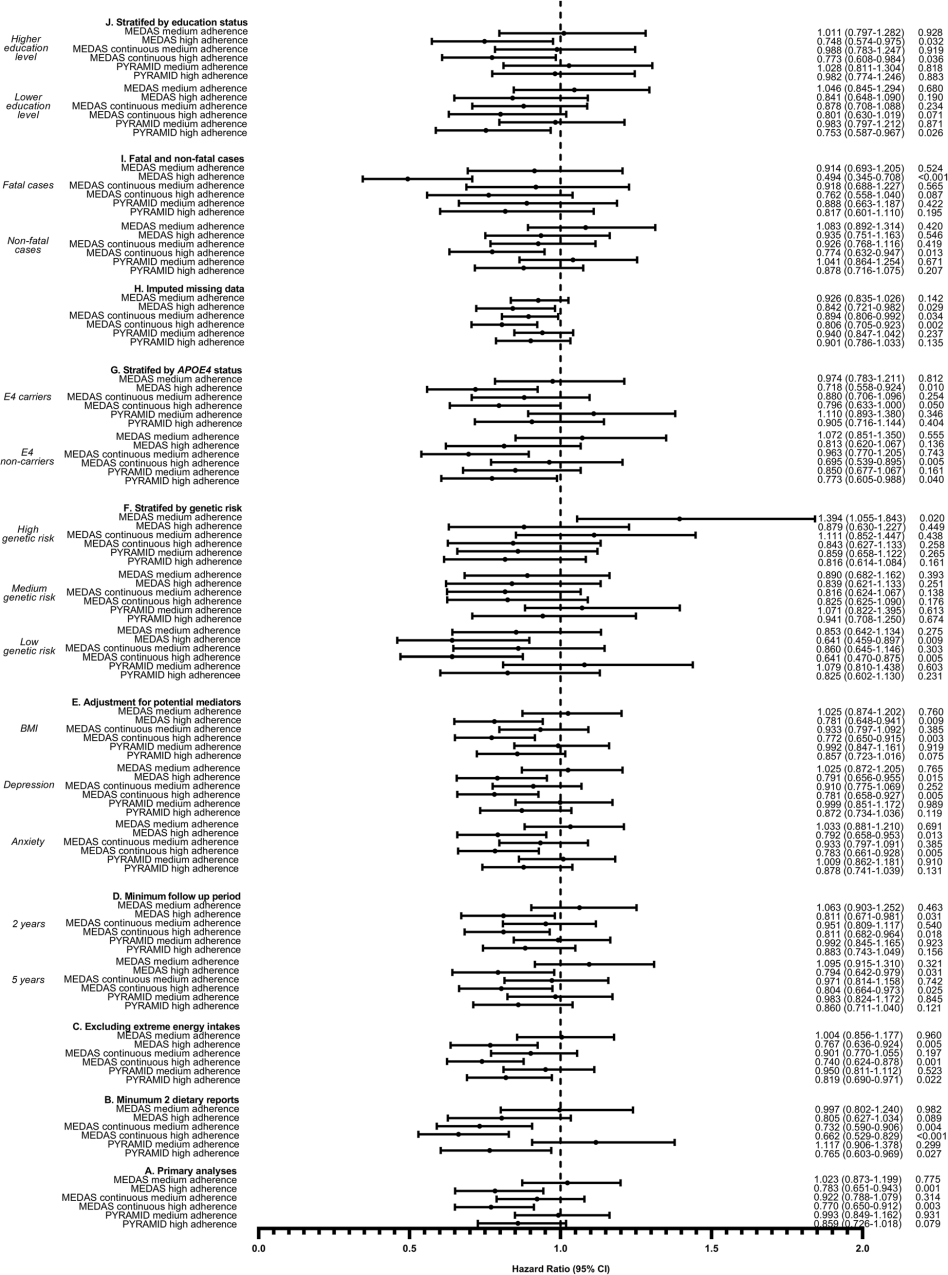
Association between MedDiet adherence and risk of dementia in sub-group and sensitivity analyses. MedDiet adherence level was split into tertiles, with the dashed line reflecting the low MedDiet adherence reference group for each MedDiet score. Analyses include: A) Primary analyses for the MEDAS, MEDAS continuous and PYRAMID scores (*n* = 60,298, including 882 dementia cases); B) Including participants with a minimum of 2 dietary reports (*n* = 38,794, including 479 dementia cases); C) After excluding participants with extreme energy intakes (*n* = 59,627, including 867 dementia cases); D) Excluding participants with less than 2 years (*n* = 59,594, including 843 dementia cases) and 5 years (*n* = 58,196, including 698 dementia cases) follow up; E) Adjusting for potential mediators, including BMI (*n* = 60,163, including 876 dementia cases), history of depression (*n* = 58,837, including 851 dementia cases), or stroke (*n* = 60,298, including 882 dementia cases); F) Stratified into low (*n* = 21,009, including 261 dementia cases), medium (*n* = 20,000, including 313 dementia cases) and high (*n* = 19,273, including 308 dementia cases) genetic risk categories; G) Stratified into *APOE4* carriers (*n* = 16,644, including 467 dementia cases) and non-carriers (*n* = 43,651, including 415 dementia cases); H) With imputed missing data (*n* = 196,335, including 5001 dementia cases); I) Restricted to fatal (*n* = 59,627, including 260 dementia cases) and non-fatal (*n* = 60,038, including 622 dementia cases) dementia cases; and J) Stratified into higher (*n* = 33,281, including 430 dementia cases) and lower (*n* = 27,007, including 452 dementia cases) education status groups

**Fig. 3:**
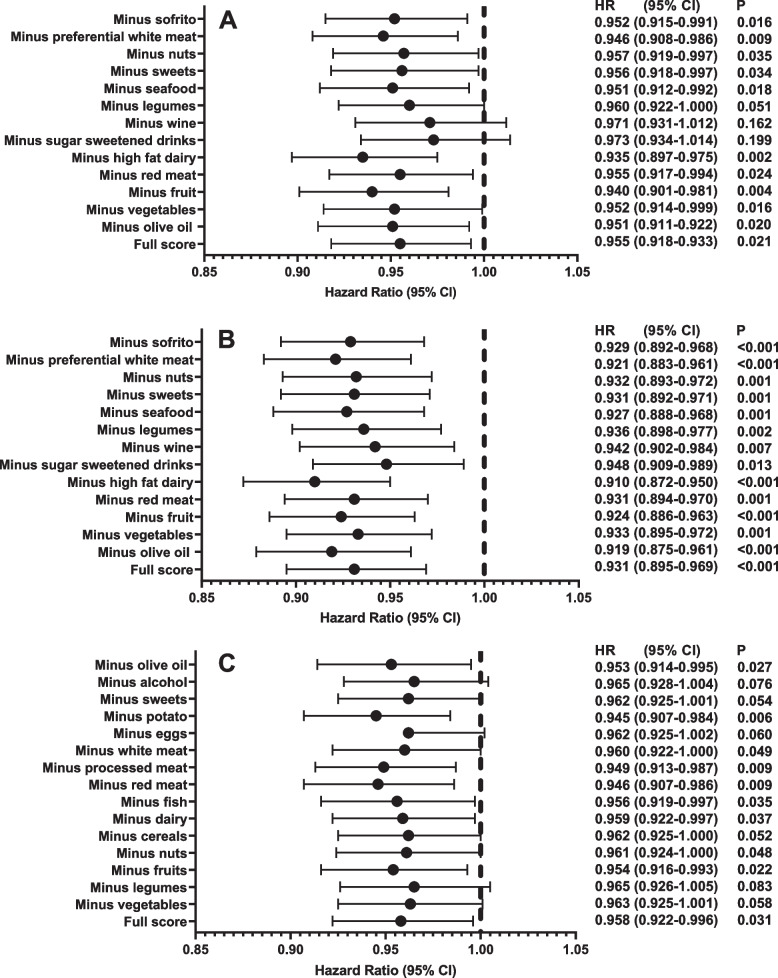
Association between MedDiet adherence defined by the MEDAS (**A**), MEDAS continuous (**B**) and PYRAMID (**C**) scores and risk of dementia (*n* = 60,298, including 882 dementia cases) after sequentially removing each MedDiet component from the total score. Hazard ratios and 95% CIs were estimated per point increased in MedDiet score

The associations between high MedDiet adherence and lower dementia risk were robust to a range of sensitivity analyses. When we used the conventional (i.e., in which points for each MedDiet component are awarded on a binary basis) rather than continuous (i.e., in which points for each MedDiet component are awarded between 0 and 1 based on proximity to the dietary targets) MEDAS score, there was a similar, albeit slightly attenuated, association between MedDiet adherence and dementia risk. Specifically, each one-point increase in MEDAS score was associated with 4.5% lower risk of dementia (HR: 0.955; 95% CI: 0.918–0.993; *p* = 0.021) and, when split into tertiles, high (HR: 0.783, 95% CI: 0.651–0.943, *p* = 0.001) but not moderate (HR: 1.023, 95% CI: 0.873–1.199, *p* = 0.775) MedDiet adherence was associated with lower dementia risk versus low MedDiet adherence (Fig. 2**)**. Results were similar when we repeated analyses for participants with a minimum of 2 dietary reports (Fig. 2 and Additional file 1, Table S4) and after excluding participants with extreme energy intakes (Fig. 2 and Additional file 1, Table S5). In analyses where MedDiet scores were derived after sequential removal of individual dietary components, the associations remained reasonably stable (Fig. 3 and Additional file 1, Table S6 and S7). Higher MedDiet adherence was associated with lower dementia risk when we repeated analyses after removing participants with less than two and less than five years of follow up to minimise risk of reverse causality (Fig. 2 and Additional file 1, Table S8), and when adjusting for potential mediators (BMI, history of depression, or stroke; Fig. 2 and Additional file 1, Table S9).

When we repeated the analyses exploring the interaction between the MedDiet adherence and polygenic risk for dementia using the conventional MEDAS score we found a significant interaction (HR: 1.042, 95% CI: 1.003–1.082; *p* = 0.035). When analyses were stratified by polygenic risk category, higher MedDiet adherence according to the MEDAS continuous scores was associated with lower dementia incidence in individuals in the lower genetic risk category only (Fig. 2 and Additional file 1, Table S10). When we repeated the analysis using the conventional MEDAS score coded on a binary basis, similar results were observed. In addition, in individuals in the higher genetic risk category, moderate MedDiet adherence according to the conventional MEDAS score was associated with higher risk for dementia (Fig. 2 and Additional file 1, Table S10). When we explored the interaction between MedDiet adherence and genetic risk defined by *APOE* genotype, no diet-gene interactions were observed (MEDAS continuous HR: 1.035; 95% CI: 0.958–1.118; *p* = 0.386; MEDAS (binary coding) HR: 0.985; 95% CI: 0.913–1.064; *p* = 0.706; PYRAMID HR: 1.054; 95% CI: 0.978–1.136; *p* = 0.167). Likewise, when analyses were stratified by *APOE* genotype, there was a similar pattern of response (i.e., higher MedDiet adherence was associated with lower HRs) in *APOE* ε4 carriers/non-carriers (Fig. 2 and Additional file 1, Table S11). Similar associations were observed when we imputed missing data (Fig. 2 and Additional file 1, Table S12), for fatal and non-fatal dementia cases (Fig. 2 and Additional file 1, Table S13), and when analyses were stratified by education status (Fig. 2 and Additional file 1, Table S14).

## Discussion

Using data from over 60,000 participants, we demonstrated that higher adherence to the MedDiet is associated with lower risk of incident all-cause dementia. Specifically, participants with the highest MedDiet adherence had 23% lower risk of developing dementia in comparison with those with the lowest level of adherence (highest vs. lowest MEDAS continuous tertiles), which was equivalent to an absolute risk difference (reduction) of 0.55%. We found no significant interaction between MedDiet adherence, defined by both the MEDAS continuous and PYRAMID scores, and polygenic risk for dementia. In addition, we found that a continuous MEDAS score was a more sensitive predictor of dementia risk when compared with a binary MEDAS or PYRAMID scores.

Previous studies exploring associations between MedDiet adherence and dementia risk have produced inconsistent findings. Indeed, a systematic review by Limongi and colleagues [9] reported lower risk of Alzheimer’s disease and all-cause dementia in four out of seven and zero out of five studies (with the other studies reporting null findings), respectively. A more recent cohort study analysis found lower risk of all-cause and non-Alzheimer’s, but not Alzheimer’s, dementia among those with higher MedDiet adherence [16]. Previous investigations have used different approaches for collecting dietary intake data (e.g., food frequency questionnaires and 24-h recall methods), and have employed various MedDiet scoring systems, each of which define adherence to this dietary pattern in distinctly different ways. Such heterogeneity could hinder efforts to interpret and compare results from different studies [9]. Indeed, although we observed broadly consistent findings across the different MedDiet scores in this study, the strength of association with dementia risk differed. Whilst diet may be an important tractable risk factor for dementia, it is not emphasised in all dementia prevention guidelines (e.g., [2]), which may reflect the lack of consistent evidence about the dietary patterns that are associated with lower dementia risk. A better understanding of the best ways to operationalize a healthy dietary pattern (including the MedDiet) will be valuable for future research studies and for the formulation of dietary guidelines to minimise dementia risk.

There is limited and inconclusive evidence about the interaction between diet (defined by MedDiet adherence or another dietary index) and genetic risk on dementia incidence [13, 18–20]. For example, higher MedDiet adherence was associated with lower dementia risk in *APOE* ε4 carriers but not non-carriers in one study [13]. In contrast, other studies have reported that higher adherence to both the MIND diet (a hybrid between the MedDiet and Dietary Approach to Stop Hypertension) [18] and a ‘healthy’ diet [19] are more protective against dementia in *APOE* ε4 non-carriers. In the present study, we found no significant interaction between polygenic risk for dementia and MedDiet adherence defined by the MEDAS continuous or PYRAMID scores in our primary analyses. Likewise, when we explored the interaction between MedDiet adherence and genetic risk defined by *APOE* genotype, there was a similar pattern of response for both *APOE* ε4 carriers/non-carriers. Thus, our findings suggest similar associations between MedDiet adherence and dementia risk irrespective of genetic risk for this condition. Nevertheless, we acknowledge a degree of uncertainty in this conclusion, given that findings were not consistent across all sensitivity analyses. Further research into the interaction between diet and genetics on dementia risk is therefore warranted.

This study has several strengths. The majority of previous studies exploring associations between MedDiet adherence and dementia risk have involved relatively small numbers of participants (*n* = 1000–6000) with limited dementia cases (*n* = 20–400) and may have lacked statistical power [9]. In contrast, our study involved a much larger cohort (*n* =  ~ 60,000) with more dementia cases (*n* = 882) than most previous investigations. We defined genetic risk for dementia using a comprehensive polygenic risk score whereas most previous studies have explored gene-diet interactions for individual genetic variants (e.g., *APOE* genotype) [13, 18–20]. A further strength of this study is that we carried out a wide range of sensitivity analyses which demonstrate the robustness of our findings. Several limitations should also be considered. Firstly, the observational design of this study precludes drawing causal inferences. Nevertheless, our findings are supported by the results from randomised controlled trials. This includes data from the Navarra [37] and Barcelona [38] cohorts of the PREDIMED trial, which demonstrated clinically meaningful benefits of a MedDiet intervention on cognitive function. A further limitation is the potential risk of reverse causality, given lower MedDiet adherence could be a consequence rather than a cause of dementia [39]. Although we did not find any evidence of reverse causality in sensitivity analyses where we excluded participants who developed dementia in the first two or five years of follow up, this does not eliminate the possibility that diet quality declined earlier in individuals who developed dementia, given the long pre-clinical phase of this condition [40, 41]. The measurement of dietary intake is a major challenge in research, and there are specific limitations related to the assessment of dietary intake in this study which should be considered when interpreting our results. We used dietary data from the Oxford WebQ, a self-administered 24-h recall method which provides results broadly comparable to those achieved via interviewer-administered 24-h recalls [25], to derive our MedDiet scores. Since multiple 24-h recalls are required to provide a ‘true’ representation of habitual diet [42], and many participants in UK Biobank completed only one or two recalls, it is possible that calculated MedDiet scores are not fully representative of the participants usual dietary intake. However, 20,348 participants repeated the touchscreen questionnaire at median 4.4 years after the initial dietary assessment. Analyses of the resulting data showed that there was moderate to substantial agreement between the responses to the dietary touchscreen questions at baseline and at the repeat visit [43]. Based on this evidence, we conclude that the estimates of dietary intake available in UK Biobank represent habitual intake and that this limits the likelihood of participant misclassification. In addition, we were unable to determine the amount of olive oil consumed from the available dietary data, which increases the risk of misclassification, as individuals who consume large amounts of olive oil may have been awarded lower scores than if we had been able to accurately quantify their intake of this MedDiet component. Nevertheless, findings from our previous research suggest that few individuals in a UK setting consume the requisite amount of olive oil to be awarded a full point for this MedDiet component [17], suggesting that this is likely to have had a limited impact on our MedDiet scores overall. Similarly, it was difficult to quantify accurately intake of sofrito, a sauce containing typically tomatoes, onions, and garlic cooked with olive oil which is popular in Mediterranean cuisine and is one of the components of the MEDAS/MEDAS continuous scores. We used self-reported intake of tomato-based sauces as the closest proxy for sofrito intake, which may have resulted in some misclassification depending upon the ingredients and preparation method. A further limitation of our work is that dementia cases were ascertained via linkage to hospital inpatient records and death registry only, which may miss some cases [44, 45]. However, previous studies have suggested good agreement with dementia ascertainment through primary care records [45]. Finally, UK Biobank participants are generally healthier and of higher socioeconomic status than the wider UK population [46] but this is unlikely to jeopardise valid assessment of exposure-disease relationships that are widely generalizable [46]. Nevertheless, since we restricted our sample to individuals of European ancestry aged ≥ 60 years at recruitment, our findings require substantiation in other populations (e.g., different ethnicities).

## Conclusions

In this large population-based prospective cohort study, higher adherence to a MedDiet was associated with reduced dementia risk. A continuous MEDAS score was the most sensitive predictor of dementia risk when compared with a binary MEDAS or PYRAMID score and could therefore be prioritised as a tool for defining MedDiet adherence in future observational studies. There was no clear evidence for an interaction with genetic risk. These results underline the importance of dietary interventions in future dementia prevention strategies regardless of genetic predisposition.

## Supplementary Information

**Additional file 1: Text S1.** Dietary assessment and creation of the MedDiet scores. **Table S1.** Components and scoring of the MEDAS and MEDAS Continuous Mediterranean diet scores. **Table S2.** Components and scoring of the PYRAMID Mediterranean diet adherence score. **Table S3.** ICD-9 and ICD-10 codes for dementia diagnosis. **Figure S1.** Participant flowchart. **Table S4.** Risk of incident dementia according to Mediterranean diet adherence, with analyses restricted to individuals with a minimum of 2 dietary reports. **Table S5.** Risk of incident dementia according to Mediterranean diet adherence excluding participants with extreme energy intakes. **Table S6.** Influence of each component of the MEDAS and MEDAS Continuous scores on dementia risk. **Table S7.** Influence of each component of the PYRAMID score on dementia risk. **Table S8.** Risk of incident dementia according to Mediterranean diet adherence, excluding participants with less than 2 years and 5 years of follow up. **Table S9**. Sensitivity analyses adjusting for potential effect mediators (BMI, Depression and stroke). **Table S10.** Association between MedDiet adherence and risk of dementia in analyses stratified by polygenic risk score tertiles. **Table S11.** Associations between MedDiet adherence and dementia incidence in APOE ε4 non-carriers and carriers. **Table S12.** Associations between MedDiet adherence and dementia incidence in analyses where missing data were imputed. **Table S13.** Risk of incident dementia according to Mediterranean diet adherence restricted to fatal and non-fatal dementia cases. **Table S14.** Risk of incident dementia according to Mediterranean diet adherence for individuals with higher and lower education levels.

## Data Availability

Data are available from UK Biobank for all bona fide researchers for health-related research in the public interest.

## Abbreviations

BMI: Body mass index
ICD: International classification of diseases
IPAQ: International physical activity questionnaire
MEDAS: Mediterranean diet adherence screener
MedDiet: Mediterranean diet
NHS: National Health Service
PREDIMED: Prevención con Dieta Mediterránea
PYRAMID: Mediterranean diet Pyramid score
SNP: Single nucleotide polymorphism
