# Supplementary material for: Mediterranean diet adherence is associated with lower dementia risk, independent of genetic predisposition: findings from the UK Biobank prospective cohort study
Source: BMC Med. 2023 Mar 14;21:81. doi: 10.1186/s12916-023-02772-3 (PMC10012551; doi:10.1186/s12916-023-02772-3)
Supplement: Supplementary file 1 — Additional file 1: Text S1. Dietary assessment and creation of the MedDiet scores. Table S1. Components and scoring of the MEDAS and MEDAS Continuous Mediterranean diet scores. Table S2. Components and scoring of the PYRAMID Mediterranean diet adherence score. Table S3. ICD-9 and ICD-10 codes for dementia diagnosis. Figure S1. Participant flowchart. Table S4. Risk of incident dementia according to Mediterranean diet adherence, with analyses restricted to individuals with a minimum of 2 dietary reports. Table S5. Risk of incident dementia according to Mediterranean diet adherence excluding participants with extreme energy intakes. Table S6. Influence of each component of the MEDAS and MEDAS Continuous scores on dementia risk. Table S7. Influence of each component of the PYRAMID score on dementia risk. Table S8. Risk of incident dementia according to Mediterranean diet adherence, excluding participants with less than 2 years and 5 years of follow up. Table S9. Sensitivity analyses adjusting for potential effect mediators (BMI, Depression and stroke). Table S10. Association between MedDiet adherence and risk of dementia in analyses stratified by polygenic risk score tertiles. Table S11. Associations between MedDiet adherence and dementia incidence in APOE ε4 non-carriers and carriers. Table S12. Associations between MedDiet adherence and dementia incidence in analyses where missing data were imputed. Table S13. Risk of incident dementia according to Mediterranean diet adherence restricted to fatal and non-fatal dementia cases. Table S14. Risk of incident dementia according to Mediterranean diet adherence for individuals with higher and lower education levels. [file 12916_2023_2772_MOESM1_ESM.docx]

**CONTENTS**

[Text S1. Dietary assessment and creation of the MedDiet scores 2](#_Toc126572634)

[Table S1. Components and scoring of the MEDAS and MEDAS Continuous Mediterranean diet adherence scales 4](#_Toc126572635)

[Table S2. Components and scoring of the PYRAMID Mediterranean diet adherence scale 6](#_Toc126572636)

[Table S3. ICD-9 and ICD-10 codes used for ascertainment of dementia diagnosis 8](#_Toc126572637)

[Figure S1. Flowchart of participants in the UK Biobank study 9](#_Toc126572638)

[Table S4. Risk of incident dementia according to Mediterranean diet adherence, with analyses restricted to individuals with a minimum of 2 dietary reports (n= 38794, including 479 dementia cases) 10](#_Toc126572639)

[Table S5. Risk of incident dementia according to Mediterranean diet adherence excluding participants with extreme energy intakes (n=59627, including 867 dementia cases) 11](#_Toc126572640)

[Table S6. Sensitivity analysis exploring the influence of each component of the MedDiet in the MEDAS and MEDAS Continuous scale on dementia risk (n=60298, including 882 dementia cases) 12](#_Toc126572641)

[Table S7. Sensitivity analysis exploring the influence of each component of the MedDiet in the PYRAMID score on dementia risk (n=60298, including 882 dementia cases). 13](#_Toc126572642)

[Table S8. Risk of incident dementia according to Mediterranean diet adherence, excluding participants with less than 2 years (n=59594, including 843 dementia cases) and 5 years (n= 58196, including 698 dementia cases) of follow up. 14](#_Toc126572643)

[Table S9. Sensitivity analyses adjusting for potential effect mediators (BMI, Depression and stroke) 15](#_Toc126572644)

[Table S10. Association between MedDiet adherence and risk of dementia in analyses stratified by polygenic risk score (PRS) tertiles 17](#_Toc126572645)

[Table S11. Associations between MedDiet adherence and dementia incidence in APOE ε4 non-carriers (low genetic risk, n=43651 including 415 dementia cases) and ε4 carriers (high genetic risk, n=16644 including 467 dementia cases). 18](#_Toc126572646)

[Table S12. Associations between MedDiet adherence and dementia incidence in analyses where missing dietary and covariate data were imputed via multiple imputations (n= 196335, including 5001 dementia cases) 19](#_Toc126572647)

[Table S13. Risk of incident dementia according to Mediterranean diet adherence restricted to fatal (n=59627, including 260 dementia cases) and non-fatal (n=60038, including 622 dementia cases) dementia cases. 20](#_Toc126572648)

[Table S14. Risk of incident dementia according to Mediterranean diet adherence for individuals with higher (college/university/other professional qualification, n=33281 including 430 dementia cases) and lower (vocational [NVQ/HND/HNC], upper secondary [A-levels], lower secondary [O-levels/GCSEs /CSEs] or no qualifications, n=27007 including 452 dementia cases) education levels 21](#_Toc126572649)

# Text S1. Dietary assessment and creation of the MedDiet scores

Dietary data from the Oxford WebQ, an extensively validated 24-hour recall tool, was used to calculate MedDiet scores in this study. Participants completed up to 5, 24-hour recalls, during which they were asked questions relating to their intake of 21 food groups during the previous day (e.g., ‘did you eat any bread or crackers yesterday?’). If an affirmative response was given to any of the questions, participants were then presented with follow-up questions enquiring about their consumption of commonly consumed foods within the food category (e.g., sliced bread, baguette, bap, bread roll etc.). Participants were required to identify the quantity of each individual food consumed using standard serving sizes (e.g., 1 slice of bread), providing information on the intake of up to 206 types of foods and 32 types of drinks.

Three different MedDiet scores were then calculated, each of which uses different criteria to quantify level of adherence to this dietary pattern. For the calculation of the MEDAS and MEDAS continuous scores, for which serving sizes are specified for each dietary target (e.g., 2 * 200 g servings of vegetables required for 1 point) the quantity of each individual food and beverage was calculated in grams by multiplying the number of participant-reported servings by a typical portion size (e.g., 2 slices of bread * 36 g = 72 g). Food items belonging to the same MedDiet category were summed. (e.g., grams of individual vegetables were summed to determine total vegetable intake etc.), before being divided by the food group portion size (e.g., total grams vegetables / 200 g = number of MEDAS-specific servings) and used to calculate the MedDiet score by comparison against the pre-defined target for each food component (e.g., 2 servings of vegetables required to achieve 1 point). For the MEDAS score, points were then awarded on a binary basis, with 0 or 1 points allocated depending on whether the participant achieved the specific dietary target. For the MEDAS continuous score, the same dietary targets were used as for the MEDAS score, but points were allocated continuously between 0 and 1 based on proximity to the dietary targets using linear equation principles. Meanwhile, for the PYRAMID score, participant self-reported servings were used to determine the level of MedDiet adherence. Servings of individual foods within a food category were weighted equally (e.g., 1 serving of broccoli is equivalent to 1 serving of carrot etc.) and, as with the MEDAS continuous score, linear equation principles were used to determine the allocation of points on a continuous basis between 0 and 1. For all three MedDiet scores, dietary data for each time point were energy adjusted to a 2000 kcal/d diet via the residuals method (28) to allow evaluation of diet quality independent of diet quantity. After excluding atypical diets, data were then averaged across all available time points for each participant prior to score creation.

As a hypothetical example, an individual consuming 2 servings of raw carrots (2 * 60 g = 120g), 1 serving of raw spinach (1 * 90 g), and 1 serving of cooked broccoli (1 * 85 g), but no other vegetables, has a total intake of 295 g (120 + 90 + 85 = 295 g) or ~ 1.5 servings (295 / 200 = 1.475) of vegetables according to the MEDAS-specific portion sizes. This individual would be awarded 0 points for this specific MedDiet food component for the MEDAS score, as they have not achieved the dietary target of 2 servings (i.e., 400 g) vegetable intake per day. By contrast, according to the MEDAS continuous score, this individual would be awarded 0.74 points (y = 0.5 * 1.475 + 0 = 0.7375 points), based around how close they are to the specific dietary target (i.e., ~3/4 of the way towards achieving the dietary target). Meanwhile, for the PYRAMID score, where the total self-reported number of servings of vegetables (4 servings) would be entered into a linear equation (y = 0.1666*x), the participant would be awarded 0.67 points (0.16666667 * 4 = 0.67). Therefore, for the same vegetable intake of 4 servings or ~295 g, a participant could be awarded 0 (MEDAS), 0.74 (MEDAS continuous) or 0.67 (PYRAMID) MedDiet points. The specific criteria used to determine score allocation for each MedDiet point is elaborated upon further in Supplementary Tables 1 and 2 below.

# Table S1. Components and scoring of the MEDAS and MEDAS Continuous Mediterranean diet adherence scales

| Food component | Contributing foods from the Oxford WebQ | MEDAS^1^ | | MEDAS continuous^2^ | |
| --- | --- | --- | --- | --- | --- |
|  |  | Servings required for 0 points | Servings required for 1 point | Servings required for 0 points | Servings required for 1 point |
| Olive oil^5^ | Type of fat/ oil used for cooking (20090) | Non-consumption | Consumption | Non-consumption | Consumption |
| Vegetables^3, 7^ | Carrot (104170), spinach (104300), broccoli (104140), cabbage/ kale (104160), sprouts (104310), courgette (104200), cauliflower (104180), parsnip (104270), turnip/ swede (104360), leek (104230), onion (104260), garlic (104220), mushroom (104250), sweet pepper (104290), side salad (104090), lettuce (104240), cucumber (104210), celery (104190), watercress (104370), fresh tomato (104340), tin tomato (104350), sweetcorn (104320), beetroot (104130), avocado (104100), mixed vegetables (104060), vegetable pieces (104070), butternut squash (104150), other veg (104380), olives (102490), coleslaw (104080), guacamole (20088), vegetables from canned soup (102540, 20108), vegetables from homemade soup (102620, 20109) | <2/d (and/or not including 1/d raw or salad) | ≥2/d (including ≥1/d raw or salad) | 0/d | ≥2/d (including ≥1/d raw or salad) |
| Fruit^3^ | Apple (104450), pear (104560), orange (104530), satsuma (104540), grapefruit (104490), banana (104460), grape (104500), melon (104520), peach/ nectarine (104550), plum (104580), berry (104470), dried fruit (104430), stewed fruit (104410), mixed fruit (104440), prune (104420), cherry (104480), mango (104510), pineapple (104570), other fruit (104590), fruit added to cereal (100880), grapefruit juice (100200), orange juice (100190) | <3/d | ≥3/d | 0/d | ≥3/d |
| Red meat^4^ | Beef (103020), pork (103030), lamb (103040), red meat from canned soup (102540, 20108), red meat from homemade soup (102620, 20109), bacon (103070), ham (103080), sausage (103010), liver (103090), meat from Scotch egg (103090) | >1/d | <1/d | ≥2/d | <1/d |
| Butter, margarine or cream^4^ | Butter/ margarine on potato (104040), baguettes with butter/ margarine (101350, 20099), baps with butter/ margarine (101390, 20100), bread rolls with butter/ margarine (101430, 20101), bread slices with butter/ margarine (101310, 20098), crackers/ crispbread with butter/ margarine (101470, 20102), oatcakes with butter/ margarine (101510, 20103), other bread with butter/ margarine (101550, 20104), butter/ margarine used in cooking (20090), cream (20088) | >1/d | <1/d | ≥2/d | <1/d |
| Sweetened or carbonated drinks^4^ | Fizzy drinks (100170), low calories drinks (100160), squash intake (100180) | >1/d | <1/d | ≥2/d | <1/d |
| Wine^3^ | Red wine (100590, 20096), rose wine (100630, 20097), white wine (100670, 20095) | <7/wk | ≥7/wk | 0/wk | ≥7/wk |
| Legumes^3^ | Peas (104280), green beans (104120), broad beans (104110), baked beans (104000), pulses (104010), tofu (103270), hummus (20088), pulses from canned soup (102540, 20108), pulses from homemade soup (102620, 20109) | <3/wk | ≥3/wk | 0/wk | ≥3/wk |
| Seafood^3^ | Battered fish (103180), breaded fish (103170), white fish (103190), oily fish (103160), shellfish (103220), other fish (103230), tinned tuna (103150), prawn (103200), lobster/ crab (103210), fish from canned soup (102540, 20108), fish from homemade soup (102620, 20109) | <3/wk | ≥3/wk | 0/wk | ≥3/wk |
| Sweets or pastries^4^ | Chocolate biscuit (102350), chocolate covered biscuit (102340), chocolate bar (102260), chocolate sweets (102310), chocolate raisins (102300), dark chocolate (102290), milk chocolate (102280), white chocolate (102270), sweet biscuits (102360), cakes (102190), cheesecake (102220), doughnut (102200), fruitcake (102180), Danish pastry (102060), sponge pudding (102210), milk based pudding (102140), other milk based pudding (102150), other desert intake (102230), soya desert intake (102170), sweets (102330), diet sweets (102320), other sweets (102380), ice cream (102120) | >2/wk | <2/wk | ≥4/wk | <2/wk |
| Nuts^3^ | Unsalted nuts (102440), salted nuts (102430), unsalted peanuts (102420), salted peanuts (102410), peanut butter (20088) | <3/wk | ≥3/wk | 0/wk | ≥3/wk |
| White meat^6^ | Poultry (103060), breaded poultry (103050), white meat from canned soup (102540, 20108), white meat from homemade soup (102620, 20109) | Less white meat than red meat | More white meat than red meat | Less white meat than red meat | More white meat than red meat |
| Sofrito^3^ | Tomato-based sauce (20088) | <2/wk | ≥2/wk | 0/wk | ≥2/wk |

^1^Scoring for the MEDAS scale was calculated as previously described (29). A score of 1 point was awarded if participants achieved a dietary target. Otherwise, participants were awarded 0 points. ^2^Scoring for the MEDAS continuous scale was carried out as described by Shannon et al. (17). This used the same dietary components as the standard MEDAS scale. However, rather than awarding points were awarded continuously between 0 and 1 based on linear equation principles. ^3^A high intake of vegetables, fruit, wine, legumes, seafood, nuts, and sofrito was recommended. For the MEDAS continuous scale, points were allocated between 0 for no consumption and 1 for meeting the recommended intake. ^4^A low intake of red meat, butter, margarine or cream, sweetened or carbonated drinks, and sweets or pastries was recommended. For the MEDAS continuous scale, points were allocated continuously between 0 points for double the recommended intake and 1 point for below the recommended intake. ^5^For olive oil individuals who reported consumption received a score of 1 point, whilst non-consumers received 0 points. As it was not possible to quantify amount of olive oil consumed, only 1 point was allocated for olive oil intake whereas the traditional MEDAS score awards points a) using olive oil as the primary cooking fat and b) for a total olive oil intake >50 ml/d. ^6^For white meat, participants were awarded a point if the total amount of white meat consumed exceeded red meat consumption. ^7^A maximum score of 0.5 points was awarded for participants who did not also consume 1 serving per day of raw vegetables or salad as part of the MEDAS continuous scale. Conversely, 0 points were awarded for participants who did not consume 1 serving per day of raw vegetables or salad as part of the MEDAS scale, irrespective of their total vegetable intake.

# Table S2. Components and scoring of the PYRAMID Mediterranean diet adherence scale

| Food component | Contributing foods from the Oxford WebQ | Recommended intake | Servings required for 0 points | Servings required for 1 point |
| --- | --- | --- | --- | --- |
| Vegetables^1^ | Carrot (104170), spinach (104300), broccoli (104140), cabbage/ kale (104160), sprouts (104310), courgette (104200), cauliflower (104180), parsnip (104270), turnip/ swede (104360), leek (104230), onion (104260), garlic (104220), mushroom (104250), sweet pepper (104290), side salad (104090), lettuce (104240), cucumber (104210), celery (104190), watercress (104370), fresh tomato (104340), tin tomato (104350), sweetcorn (104320), beetroot (104130), avocado (104100), mixed vegetables (104060), vegetable pieces (104070), butternut squash (104150), other veg (104380), olives (102490), coleslaw (104080), guacamole (20088), vegetables from canned soup (102540, 20108), vegetables from homemade soup (102620, 20109) | ≥6/d | 0/d | ≥6/d |
| Legumes^1^ | Peas (104280), green beans (104120), broad beans (104110), baked beans (104000), pulses (104010), tofu (103270), hummus (20088), pulses from canned soup (102540, 20108), pulses from homemade soup (102620, 20109) | ≥2/wk | 0/wk | ≥2/wk |
| Fruits^2^ | Apple (104450), pear (104560), orange (104530), satsuma (104540), grapefruit (104490), banana (104460), grape (104500), melon (104520), peach/ nectarine (104550), plum (104580), berry (104470), dried fruit (104430), stewed fruit (104410), mixed fruit (104440), prune (104420), cherry (104480), mango (104510), pineapple (104570), other fruit (104590), fruit added to cereal (100880) | 3-6/d | 0/d | 3-6/d |
| Nuts^2^ | Unsalted nuts (102440), salted nuts (102430), unsalted peanuts (102420), salted peanuts (102410), peanut butter (20088) | 1-2/d | 0/d | 1-2/d |
| Cereals^2^ | Sliced bread (100950), bread roll (101160), baguette (101020), bap (101090), other bread (101270), crisp bread (101250), porridge (100770), muesli (100800), oat crunch (100810), sweetened cereal (100820), plain cereal (100830), bran cereal (100840), whole wheat cereal (100850), other cereal (100860), white rice (102730), brown rice (102740), white pasta (102710), wholemeal pasta (102720), couscous (102770), cereal bar (102370), other grain (102780) | 3-6/d | 0/d | 3-6/d |
| Dairy^2^ | Yogurt (102090), low fat hard cheese (102810), hard cheese (102820), low fat cheese spread (102850), cheese spread (102860), soft cheese (102830), goat cheese (102900), blue cheese (102840), feta (102880), mozzarella (102890), other cheese (102910), cottage cheese (102870), milk (100520), flavoured milk (100530), added milk instant coffee (100260, 100250), added milk filtered coffee (100280, 100270), added milk espresso (100320, 100310), added milk other coffee (100350, 100330), added milk standard tea (100460, 100400), added milk rooibos tea (100480, 100410), dairy smoothie (100230), latte (100300), cappuccino (100290), milk added to cereal (100890, 100770, 100800, 100810, 100820, 100830, 100840, 100850, 100860), type of milk used (100920) | 2/d | 0/d | 1.5-2.5/d |
| Fish^1^ | Battered fish (103180), breaded fish (103170), white fish (103190), oily fish (103160), shellfish (103220), other fish (103230), tinned tuna (103150), prawn (103200), lobster/ crab (103210), fish from canned soup (102540, 20108), fish from homemade soup (102620, 20109) | ≥2/wk | 0/wk | ≥2/wk |
| Red meat^3^ | Beef (103020), pork (103030), lamb (103040), red meat from canned soup (102540, 20108), red meat from homemade soup (102620, 20109) | ˂2/wk | ≥4/wk | ˂2/wk |
| Processed meat^3^ | Bacon (103070), ham (103080), sausage (103010), liver (103090), meat from Scotch egg (103090) | ≤1/wk | ≥2/wk | ≤1/wk |
| White meat^2^ | Poultry (103060), breaded poultry (103050), white meat from canned soup (102540, 20108), white meat from homemade soup (102620, 20109) | 2/wk | 0/wk | 1.5-2.5/wk |
| Egg^2^ | Whole egg (102940), omelette (102950), egg sandwiches (102960), scotch egg (102970), other egg (102980) | 2-4/wk | 0/wk | 2-4/wk |
| Potato^3^ | Boiled/ baked potato (104030), mashed potato (104050), fried potato (104020) | ≤3/wk | ≥6/wk | ≤3/wk |
| Sweets^3^ | Chocolate biscuit (102350), chocolate covered biscuit (102340), chocolate bar (102260), chocolate sweets (102310), chocolate raisins (102300), dark chocolate (102290), milk chocolate (102280), white chocolate (102270), sweet biscuits (102360), cakes (102190), cheesecake (102220), doughnut (102200), fruitcake (102180), Danish pastry (102060), sponge pudding (102210), milk based pudding (102140), other milk based pudding (102150), other desert intake (102230), soya desert intake (102170), sweets (102330), diet sweets (102320), other sweets (102380), ice cream (102120), fizzy drinks (100170), low calories drinks (100160), squash intake (100180), sugar added to tea (100490, 100400, 100410), sugar added to coffee (100370, 100250, 100270, 100310, 100330, 100300, 100290), sugar added to cereal (100900, 100770, 100800, 100810, 100820, 100830, 100840, 100850, 100860) | ≤2/wk | ≥4/wk | ≤2/wk |
| Alcohol^4^ | Red wine (100590, 20096), rose wine (100630, 20097), white wine (100670, 20095), beer cider (100710), fortified wine (100720), spirits (100730), other alcohol (100740) | 2/d for men  1/d for women | Men = ≥4/d  Women = ≥2/d | Men = 1.5-2.5/d  Women = 0.5-1.5/d |
| Olive oil^5^ | Type of fat/ oil used for cooking (20090) | Principal source of dietary lipids | Non-consumption | Consumption |

Scoring for the PYRAMID scale was calculated using the methods proposed by Tong et al. (27). ^1^A high intake of vegetables, legumes, and fish was recommended. Points were allocated continuously between 0 (no consumption) and 1 (achieving or exceeding the recommended intake). ^2^A Moderate intake of fruits, nuts, cereals, dairy, white meat, and eggs was recommended. Points were allocated continuously between 0 (no consumption) and 1 (achieving an intake within the recommended level). Overconsumption (consuming an amount double the mid-point of the recommended intake), was penalised and received a maximum of 0.5 points, with points allocated proportionally between the recommended level and the penalty point. ^3^A low intake of red meat, processed meat, potato, and sweets was recommended. Points were allocated continuously between 0 points (double the recommended intake) and 1 point (below the recommended intake). ^4^Separate recommendations were provided for alcohol consumption in men and women. Consumption within the recommended intake received 1 point. Conversely, overconsumption received 0 points, and non-consumption received a score of 0.5 points. Points were allocated proportionally between 0.5 points and 1 point for intake between non-consumption and the recommended level for alcohol intake. ^5^For olive oil, individuals who reported consumption received a score of 1 point, whilst non-consumers received 0 points.

# Table S3. ICD-9 and ICD-10 codes used for ascertainment of dementia diagnosis

| Dementia sub-type | ICD-9 codes | ICD-10 codes |
| --- | --- | --- |
| Alzheimer’s | 331.0 | F00, F00.0, F00.1, F00.2, F00.9, G30, G30.0, G30.1, G30.8, G30.9 |
| Vascular dementia | 290.4 | F01, F01.0, F01.1, F01.2, F01.3, F01.8, F01.9, I67.3 |
| Frontotemporal dementia | 331.1 | F02.0, G31.0 |
| Other codes for all-cause dementia | 290.2, 290.3, 291.2, 294.1, 331.2, 331.5 | A81.0, F02, F02.1, F02.2, F02.3, F02.4, F02.8, F03, F05.1, F10.6, G31.1, G31.8 |

# **Figure S1.** Flowchart of participants in the UK Biobank study

# Table S4. Risk of incident dementia according to Mediterranean diet adherence, with analyses restricted to individuals with a minimum of 2 dietary reports (n= 38794, including 479 dementia cases)

|  | MEDAS | | | MEDAS continuous | | | PYRAMID | | |
| --- | --- | --- | --- | --- | --- | --- | --- | --- | --- |
|  | Low  (n=9603) | Medium  (n=16932) | High  (n=12259) | Low  (n=12647) | Medium  (n=12935) | High  (n=13212) | Low  (n=12687) | Medium  (n=12997) | High  (n=13110) |
| HR (95% CI) | 1 (reference) | 0.997 (0.802 – 1.240) | 0.805 (0.627-1.034) | 1 (reference) | 0.732 (0.590 – 0.906) | 0.662 (0.529 – 0.829) | 1 (reference) | 1.117 (0.906 – 1.378) | 0.765 (0.603 – 0.969) |
| P value | - | 0.982 | 0.089 | - | **0.004** | **<0.001** | - | 0.299 | **0.027** |
| Continuous HR (95% CI) | 0.946 (0.897 – 0.999) | | | 0.912 (0.862 – 0.964) | | | 0.947 (0.899 – 0.999) | | |
| P value | **0.044** | | | **0.001** | | | **0.044** | | |

# Table S5. Risk of incident dementia according to Mediterranean diet adherence excluding participants with extreme energy intakes (n=59627, including 867 dementia cases)

|  | MEDAS | | | MEDAS continuous | | | PYRAMID | | |
| --- | --- | --- | --- | --- | --- | --- | --- | --- | --- |
|  | Low  (n=15189) | Medium  (n=25868) | High  (n=18570) | Low  (n=19272) | Medium  (n=19836) | High  (n=20519) | Low  (n=19397) | Medium  (n=19941) | High  (n=20289) |
| HR (95% CI) | 1 (reference) | 1.004 (0.856-1.177) | 0.767 (0.636-0.924) | 1 (reference) | 0.901 (0.770 – 1.055) | 0.740 (0.624 – 0.878) | 1 (reference) | 0.950 (0.811 – 1.112) | 0.819 (0.690 – 0.971) |
| P value | - | 0.960 | **0.005** | - | 0.197 | **0.001** | - | 0.523 | **0.022** |
| Continuous HR (95% CI) | 0.954 (0.917 – 0.992) | | | 0.924 (0.888 – 0.962) | | | 0.951 (0.915 – 0.989) | | |
| P value | **0.019** | | | **<0.001** | | | **0.011** | | |

# Table S6. Sensitivity analysis exploring the influence of each component of the MedDiet in the MEDAS and MEDAS Continuous scale on dementia risk (n=60298, including 882 dementia cases)

|  | **MEDAS** | | **MEDAS Continuous** | |
| --- | --- | --- | --- | --- |
| **Component** |  | |  | |
|  | **HR (95%CI)** | **P** | **HR (95% CI)** | **P** |
| Full score | 0.955 (0.918 – 0.993) | **0.021** | 0.931 (0.895 – 0.969) | **<0.001** |
| Minus olive oil  (consumers vs. non-consumers) | 0.951 (0.911 – 0.992) | **0.020** | 0.919 (0.879 – 0.961) | **<0.001** |
| Minus vegetables | 0.952 (0.914 – 0.999) | **0.016** | 0.933 (0.895 – 0.972) | **0.001** |
| Minus fruit | 0.940 (0.901 – 0.981) | **0.004** | 0.924 (0.886 – 0.963) | **<0.001** |
| Minus red meat | 0.955 (0.917 – 0.994) | **0.024** | 0.931 (0.894 – 0.970) | **0.001** |
| Minus high fat dairy | 0.935 (0.897 – 0.975) | **0.002** | 0.910 (0.872 – 0.950) | **<0.001** |
| Minus sugar sweetened drinks | 0.973 (0.934 – 1.014) | 0.199 | 0.948 (0.909 – 0.989) | **0.013** |
| Minus wine | 0.971 (0.931 – 1.012) | 0.162 | 0.942 (0.902 – 0.984) | **0.007** |
| Minus legumes | 0.960 (0.922 – 1.000) | 0.051 | 0.936 (0.898 – 0.977) | **0.002** |
| Minus seafood | 0.951 (0.912 – 0.992) | **0.018** | 0.927 (0.888 – 0.968) | **0.001** |
| Minus sweets | 0.956 (0.918 – 0.997) | **0.034** | 0.931 (0.892 – 0.971) | **0.001** |
| Minus nuts | 0.957 (0.919 – 0.997) | **0.035** | 0.932 (0.893 – 0.972) | **0.001** |
| Minus preferential white meat | 0.946 (0.908 – 0.986) | **0.009** | 0.921 (0.883 – 0.961) | **<0.001** |
| Minus sofrito | 0.952 (0.915 – 0.991) | **0.016** | 0.929 (0.892 – 0.968) | **<0.001** |

# Table S7. Sensitivity analysis exploring the influence of each component of the MedDiet in the PYRAMID score on dementia risk (n=60298, including 882 dementia cases).

| **Component** |  | |
| --- | --- | --- |
|  | **HR (95% CI)** | **P** |
| Full score | 0.958 (0.922 – 0.996) | **0.031** |
| Minus vegetables | 0.963 (0.925 – 1.001) | 0.058 |
| Minus legumes | 0.965 (0.926 – 1.005) | 0.083 |
| Minus fruits | 0.954 (0.916 – 0.993) | **0.022** |
| Minus nuts | 0.961 (0.924 – 1.000) | **0.048** |
| Minus cereals | 0.962 (0.925 – 1.000) | 0.052 |
| Minus dairy | 0.959 (0.922 – 0.997) | **0.037** |
| Minus fish | 0.956 (0.916 – 0.997) | **0.035** |
| Minus red meat | 0.946 (0.907 – 0.986) | **0.009** |
| Minus processed meat | 0.949 (0.913 – 0.987) | **0.009** |
| Minus white meat | 0.960 (0.922 – 1.000) | **0.049** |
| Minus eggs | 0.962 (0.925 – 1.002) | 0.060 |
| Minus potato | 0.945 (0.907 – 0.984) | **0.006** |
| Minus sweets | 0.962 (0.925 – 1.001) | 0.054 |
| Minus alcohol | 0.965 (0.928 – 1.004) | 0.076 |
| Minus olive oil  (consumers vs. non-consumers) | 0.953 (0.914 – 0.995) | **0.027** |

# Table S8. Risk of incident dementia according to Mediterranean diet adherence, excluding participants with less than 2 years (n=59594, including 843 dementia cases) and 5 years (n= 58196, including 698 dementia cases) of follow up.

| **Removal of participants with < 2 years follow up** | | | | | | | | | |
| --- | --- | --- | --- | --- | --- | --- | --- | --- | --- |
|  | MEDAS | | | MEDAS continuous | | | PYRAMID | | |
|  | Low  (n=15087) | Medium  (n=25841) | High  (n=18666) | Low  (n=19092) | Medium  (n=19893) | High  (n=20609) | Low  (n=19336) | Medium  (n=19890) | High  (n=20368) |
| HR (95% CI) | 1 (reference) | 1.063 (0.903 – 1.252) | 0.811 (0.671 – 0.981) | 1 (reference) | 0.951 (0.809 – 1.117) | 0.811 (0.682 – 0.964) | 1 (reference) | 0.992 (0.845 – 1.165) | 0.883 (0.743 – 1.049) |
| P value | - | 0.463 | **0.031** | - | 0.540 | **0.018** | - | 0.923 | 0.156 |
| Continuous HR (95% CI) | 0.966 (0.928 – 1.006) | | | 0.946 (0.908 – 0.985) | | | 0.968 (0.931 – 1.007) | | |
| P value | 0.092 | | | **0.007** | | | 0.105 | | |
| **Removal of participants with < 5 years follow up** | | | | | | | | | |
|  | MEDAS | | | MEDAS continuous | | | PYRAMID | | |
|  | Low  (n=14661) | Medium  (n=25243) | High  (n=18292) | Low  (n=18561) | Medium  (n=19439) | High  (n=20196) | Low  (n=18825) | Medium  (n=19438) | High  (n=19933) |
| HR (95% CI) | 1 (reference) | 1.095 (0.915-1.310) | 0.793 (0.642-0.979) | 1 (reference) | 0.971 (0.814-1.158) | 0.804 (0.664-0.973) | 1 (reference) | 0.983 (0.824-1.172) | 0.860 (0.711-1.040) |
| P value | - | 0.321 | **0.031** | - | 0.742 | **0.025** | - | 0.845 | 0.121 |
| Continuous HR (95% CI) | 0.961 (0.919-1.004) | | | 0.942 (0.900-0.985) | | | 0.971 (0.930-1.014) | | |
| P value | 0.073 | | | **0.009** | | | 0.187 | | |

# Table S9. Sensitivity analyses adjusting for potential effect mediators (BMI, Depression and stroke)

| **BMI** (n=60163, including 876 dementia cases) | | | | | | | | | |
| --- | --- | --- | --- | --- | --- | --- | --- | --- | --- |
|  | MEDAS | | | MEDAS continuous | | | PYRAMID | | |
|  | Low  (n=15279) | Medium  (n=26091) | High  (n=18793) | Low  (n=19345) | Medium  (n=20079) | High  (n=20739) | Low  (n=19569) | Medium  (n=20073) | High  (n=20521) |
| HR (95% CI) | 1 (reference) | 1.025 (0.874 – 1.202) | 0.781 (0.648 – 0.941) | 1 (reference) | 0.933 (0.797 – 1.092) | 0.772 (0.650 – 0.915) | 1 (reference) | 0.992 (0.847 – 1.161) | 0.857 (0.723 – 1.016) |
| P value | - | 0.760 | **0.009** | - | 0.385 | **0.003** | - | 0.919 | 0.075 |
| Continuous HR (95% CI) | 0.954 (0.917 – 0.992) | | | 0.931 (0.894 – 0.969) | | | 0.959 (0.923 – 0.997) | | |
| P value | **0.019** | | | **0.001** | | | **0.034** | | |
| **Depression** (n=58837, including 851 dementia cases) | | | | | | | | | |
|  | MEDAS | | | MEDAS continuous | | | PYRAMID | | |
|  | Low  (n=14839) | Medium  (n=25533) | High  (n=18465) | Low  (n=18801) | Medium  (n=19665) | High  (n=20371) | Low  (n=19086) | Medium  (n=19634) | High  (n=20117) |
| HR (95% CI) | 1 (reference) | 1.025 (0.872 – 1.205) | 0.791 (0.656 – 0.955) | 1 (reference) | 0.910 (0.775 – 1.069) | 0.781 (0.658 – 0.927) | 1 (reference) | 0.999 (0.851 – 1.172) | 0.872 (0.734 – 1.036) |
| P value | - | 0.765 | **0.015** | - | 0.252 | **0.005** | - | 0.989 | 0.119 |
| Continuous HR (95% CI) | 0.957 (0.920 – 0.996) | | | 0.934 (0.897 – 0.973) | | | 0.960 (0.923 – 0.998) | | |
| P value | **0.031** | | | **0.001** | | | **0.040** | | |
| **Stroke** (n=60298, including 882 dementia cases) | | | | | | | | | |
|  | MEDAS | | | MEDAS continuous | | | PYRAMID | | |
|  | Low  (n=15319) | Medium  (n = 26143) | High  (n = 18836) | Low  (n=19393) | Medium  (n = 20120) | High  (n=20785) | Low  (n=19613) | Medium  (n=20122) | High  (n=20563) |
| HR (95% CI) | 1 (reference) | 1.033 (0.881 – 1.210) | 0.792 (0.658 – 0.953) | 1 (reference) | 0.933 (0.797 – 1.091) | 0.783 (0.661 – 0.928) | 1 (reference) | 1.009 (0.862 – 1.181) | 0.878 (0.741 – 1.039) |
| P value | - | 0.691 | **0.013** | - | 0.385 | **0.005** | - | 0.910 | 0.131 |
| Continuous HR (95% CI) | 0.957 (0.921 – 0.996) | | | 0.935 (0.899 – 0.974) | | | 0.963 (0.926 – 1.000) | | |
| P value | **0.029** | | | **0.001** | | | 0.053 | | |

# Table S10. Association between MedDiet adherence and risk of dementia in analyses stratified by polygenic risk score (PRS) tertiles

|  | Low PRS  (n=21009, dementia cases = 261) | | | Medium PRS  (n=20,000, dementia cases = 313) | | | High PRS  (n=19273, dementia cases = 308) | | |
| --- | --- | --- | --- | --- | --- | --- | --- | --- | --- |
| **MEDAS score** | | | | | | | | | |
| MedDiet adherence | Low  (n=5286) | Med  (n=9076) | High  (n=6647) | Low  (n=5116) | Med  (n=8691) | High  (n=6193) | Low  (n=4912) | Med  (n=8369) | High  (n=5992) |
| HR (95%CI) | 1 (reference) | 0.853 (0.642-1.134) | 0.641 (0.459 – 0.897) | 1 (reference) | 0.890 (0.682 – 1.162) | 0.839 (0.621 – 1.133) | 1 (reference) | 1.394 (1.055 – 1.843) | 0.879 (0.630 – 1.227) |
| P value | - | 0.275 | **0.009** | - | 0.393 | 0.251 | - | **0.020** | 0.449 |
| **MEDAS Continuous score** | | | | | | | | | |
| MedDiet adherence | Low  (n=6685) | Med  (n=6801) | High  (n=7523) | Low  (n=6473) | Med  (n=6716) | High  (n=6811) | Low  (n=6228) | Med  (n=6599) | High  (n=6446) |
| HR (95%CI) | 1 (reference) | 0.860 (0.645 – 1.146) | 0.641 (0.470 – 0.875) | 1 (reference) | 0.816 (0.624 – 1.067) | 0.825 (0.625 – 1.090) | 1 (reference) | 1.111 (0.852 – 1.447) | 0.843 (0.627 – 1.133) |
| P value | - | 0.303 | **0.005** | - | 0.138 | 0.176 | - | 0.438 | 0.258 |
| **PYRAMID score** | | | | | | | | | |
| MedDiet adherence | Low  (n=6732) | Med  (n=6954) | High  (n=7323) | Low  (n=6536) | Med  (n=6639) | High  (n=6825) | Low  (n=6340) | Med  (n=6522) | High  (n=6411) |
| HR (95%CI) | 1 (reference) | 1.079 (0.810 – 1.438) | 0.825 (0.602 – 1.130) | 1 (reference) | 1.071 (0.822 – 1.395) | 0.941 (0.708 – 1.250) | 1 (reference) | 0.859 (0.658 – 1.122) | 0.816 (0.614 – 1.084) |
| P value | - | 0.603 | 0.231 | - | 0.613 | 0.674 | - | 0.265 | 0.161 |

# Table S11. Associations between MedDiet adherence and dementia incidence in APOE ε4 non-carriers (low genetic risk, n=43651 including 415 dementia cases) and ε4 carriers (high genetic risk, n=16644 including 467 dementia cases).

|  | ε4 non-carriers (low genetic risk) | | | ε4 carriers (high genetic risk) | | |
| --- | --- | --- | --- | --- | --- | --- |
|  | MEDAS | | | MEDAS | | |
|  | Low  (n=11187) | Medium  (n=18959) | High  (n=13505) | Low  (n=4130) | Medium  (n=7184) | High  (n=5330) |
| HR (95%CI) | 1 (reference) | 1.072 (0.851 – 1.350) | 0.813 (0.620 – 1.067) | 1 (reference) | 0.974 (0.783 – 1.211) | 0.718 (0.558 – 0.924) |
| P value | - | 0.555 | 0.136 | - | 0.812 | **0.010** |
|  | MEDAS continuous | | | MEDAS continuous | | |
|  | Low  (n=14147) | Medium  (n=14568) | High  (n=14936) | Low  (n=5244) | Medium  (n=5552) | High  (n=5848) |
| MEDAS CONTINUOUS  HR (95%CI) | 1 (reference) | 0.963 (0.770 – 1.205) | 0.695 (0.539-0.895) | 1 (reference) | 0.880 (0.706 – 1.096) | 0.796 (0.633 – 1.000) |
| P value | - | 0.743 | **0.005** | - | 0.254 | 0.050 |
|  | PYRAMID | | | PYRAMID | | |
|  | Low  (n=14268) | Medium  (n=14571) | High  (n=14812) | Low  (n=5345) | Medium  (n=5549) | High  (n=5750) |
| PYRAMID  HR (95%CI) | 1 (reference) | 0.850 (0.677 – 1.067) | 0.773 (0.605 – 0.988) | 1 (reference) | 1.110 (0.893 – 1.380) | 0.905 (0.716 – 1.144) |
| P value | - | 0.161 | **0.040** | - | 0.346 | 0.404 |

# Table S12. Associations between MedDiet adherence and dementia incidence in analyses where missing dietary and covariate data were imputed via multiple imputations (n= 196335, including 5001 dementia cases)

|  | MEDAS | | | MEDAS continuous | | | PYRAMID | | |
| --- | --- | --- | --- | --- | --- | --- | --- | --- | --- |
|  | Low | Medium | High | Low | Medium | High | Low | Medium | High |
| HR (95% CI) | 1 (reference) | 0.926 (0.835 – 1.026) | 0.842 (0.721 – 0.982) | 1 (reference) | 0.894 (0.806 – 0.992) | 0.806 (0.705 – 0.923) | 1 (reference) | 0.940 (0.847 – 1.042) | 0.901 (0.786 – 1.033) |
| P value | - | 0.142 | **0.029** | - | **0.034** | **0.002** | - | 0.237 | 0.135 |
| Continuous HR (95% CI) | 0.963 (0.930 – 0.998) | | | 0.941 (0.909 – 0.975) | | | 0.971 (0.940 – 1.003) | | |
| P value | **0.037** | | | **0.001** | | | 0.072 | | |
|  | MEDAS * genetic risk | | | MEDAS continuous * genetic risk | | | PYRAMID * genetic risk | | |
| Continuous HR (95% CI) | 1.008 (0.986 – 1.031) | | | 1.009 (0.987 – 1.031) | | | 1.008 (0.988 – 1.028) | | |
| P for interaction | 0.460 | | | 0.412 | | | 0.449 | | |

Missing dietary and covariate data were imputed using the SPSS multiple imputations procedure, with 70 imputations and 20 iterations. All analytic variables were included in the model. In addition, abbreviated MedDiet scores, created using data from the UK Biobank touchscreen questionnaire, were included to provide supplementary information on dietary intake.

# Table S13. Risk of incident dementia according to Mediterranean diet adherence restricted to fatal (n=59627, including 260 dementia cases) and non-fatal (n=60038, including 622 dementia cases) dementia cases.

| **Fatal dementia cases** | | | | | | | | | |
| --- | --- | --- | --- | --- | --- | --- | --- | --- | --- |
|  | MEDAS | | | MEDAS continuous | | | PYRAMID | | |
|  | Low  (n=15140) | Medium  (n=25832) | High  (n=18655) | Low  (n=19133) | Medium  (n=19893) | High  (n=20601) | Low  (n=19368) | Medium  (n=19881) | High  (n=20378) |
| HR (95% CI) | 1 (reference) | 0.914 (0.693-1.205) | 0.494 (0.345-0.708) | 1 (reference) | 0.918 (0.688-1.227) | 0.762 (0.558-1.040) | 1 (reference) | 0.888 (0.663-1.187) | 0.817 (0.601-1.110) |
| P value | - | 0.524 | **<0.001** | - | 0.565 | 0.087 | - | 0.422 | 0.195 |
| Continuous HR (95% CI) | 0.883 (0.821-0.950) | | | 0.900 (0.836-0.969) | | | 0.952 (0.887-1.021) | | |
| P value | **<0.001** | | | **0.005** | | | 0.167 | | |
| **Non-fatal dementia cases** | | | | | | | | | |
|  | MEDAS | | | MEDAS continuous | | | PYRAMID | | |
|  | Low  (n=15235) | Medium  (n=26015) | High  (n=18788) | Low  (n=19295) | Medium  (n=20031) | High  (n=20712) | Low  (n=19513) | Medium  (n=20037) | High  (n=20488) |
| HR (95% CI) | 1 (reference) | 1.083 (0.892-1.314) | 0.935 (0.751-1.163) | 1 (reference) | 0.926 (0.768-1.116) | 0.774 (0.632-0.947) | 1 (reference) | 1.041 (0.864-1.254) | 0.878 (0.716-1.075) |
| P value | - | 0.420 | 0.546 | - | 0.419 | **0.013** | - | 0.671 | 0.207 |
| Continuous HR (95% CI) | 0.986 (0.941-1.032) | | | 0.944 (0.901-0.991) | | | 0.961 (0.918-1.006) | | |
| P value | 0.539 | | | **0.019** | | | 0.092 | | |

# Table S14. Risk of incident dementia according to Mediterranean diet adherence for individuals with higher (college/university/other professional qualification, n=33281 including 430 dementia cases) and lower (vocational [NVQ/HND/HNC], upper secondary [A-levels], lower secondary [O-levels/GCSEs /CSEs] or no qualifications, n=27007 including 452 dementia cases) education levels

| **Higher education level** | | | | | | | | | |
| --- | --- | --- | --- | --- | --- | --- | --- | --- | --- |
|  | MEDAS | | | MEDAS continuous | | | PYRAMID | | |
|  | Low  (n=7109) | Medium  (n=14314) | High  (n=11858) | Low  (n=8794) | Medium  (n=11160) | High  (n=13327) | Low  (n=9168) | Medium  (n=11022) | High  (n=13091) |
| HR (95% CI) | 1 (reference) | 1.011 (0.797-1.282) | 0.748 (0.574-0.975) | 1 (reference) | 0.988 (0.783-1.247) | 0.773 (0.608-0.984) | 1 (reference) | 1.028 (0.811-1.304) | 0.982 (0.774-1.246) |
| P value | - | 0.928 | **0.032** | - | 0.919 | **0.036** | - | 0.818 | 0.883 |
| Continuous HR (95% CI) | 0.936 (0.885-0.989) | | | 0.920 (0.869-0.974) | | | 0.977 (0.926-1.032) | | |
| P value | **0.019** | | | **0.004** | | | 0.403 | | |
| **Lower education level** | | | | | | | | | |
|  | MEDAS | | | MEDAS continuous | | | PYRAMID | | |
|  | Low  (n=8205) | Medium  (n=11826) | High  (n=6976) | Low  (n=10594) | Medium  (n=8958) | High  (n=7455) | Low  (n=10441) | Medium  (n=9097) | High  (n=7469) |
| HR (95% CI) | 1 (reference) | 1.046 (0.845-1.294) | 0.841 (0.648-1.090) | 1 (reference) | 0.878 (0.708-1.088) | 0.801 (0.630-1.019) | 1 (reference) | 0.983 (0.797-1.212) | 0.753 (0.587-0.967) |
| P value | - | 0.680 | 0.190 | - | 0.234 | 0.071 | - | 0.871 | **0.026** |
| Continuous HR (95% CI) | 0.978 (0.926-1.034) | | | 0.948 (0.896-1.002) | | | 0.945 (0.894-0.997) | | |
| P value | 0.433 | | | 0.061 | | | **0.040** | | |
